# Deciphering the intricate hierarchical gene regulatory network: unraveling multi-level regulation and modifications driving secondary cell wall formation

**DOI:** 10.1093/hr/uhad281

**Published:** 2023-12-19

**Authors:** Zhigang Wei, Hairong Wei

**Affiliations:** Engineering Research Center of Agricultural Microbiology Technology, Ministhry of Education & Heilongjiang Provincial Key Laboratory of Plant Genetic Engineering and Biological Fermentation Engineering for Cold Region & Key Laboratory of Molecular Biology, College of Heilongjiang Province & School of Life Sciences, Heilongjiang University, Harbin 150080, China; College of Forest Resources and Environmental Science, Michigan Technological University, Houghton, MI 49931, USA

## Abstract

Wood quality is predominantly determined by the amount and the composition of secondary cell walls (SCWs). Consequently, unraveling the molecular regulatory mechanisms governing SCW formation is of paramount importance for genetic engineering aimed at enhancing wood properties. Although SCW formation is known to be governed by a hierarchical gene regulatory network (HGRN), our understanding of how a HGRN operates and regulates the formation of heterogeneous SCWs for plant development and adaption to ever-changing environment remains limited. In this review, we examined the HGRNs governing SCW formation and highlighted the significant key differences between herbaceous Arabidopsis and woody plant poplar. We clarified many confusions in existing literatures regarding the HGRNs and their orthologous gene names and functions. Additionally, we revealed many network motifs including feed-forward loops, feed-back loops, and negative and positive autoregulation in the HGRNs. We also conducted a thorough review of post-transcriptional and post-translational aspects, protein–protein interactions, and epigenetic modifications of the HGRNs. Furthermore, we summarized how the HGRNs respond to environmental factors and cues, influencing SCW biosynthesis through regulatory cascades, including many regulatory chains, wiring regulations, and network motifs. Finally, we highlighted the future research directions for gaining a further understanding of molecular regulatory mechanisms underlying SCW formation.

## Introduction

Wood formation, also known as xylogenesis, refers to the process through which plants produce and develop woody tissues, especially secondary xylem, which plays crucial role in providing structural support and facilitating transporting water and nutrients throughout plants [1]. The pace and characteristics of wood formation hold substantial sway over a plant's overall growth and development [2]. Both horticulturists and foresters often aim to optimize wood formation to ensure that plants have sturdy stems and branches because proper wood development is essential for supporting the weights of fruits, flowers, and foliage. In horticulture practices, pruning and training techniques have been frequently used to manipulate wood formation to maximize the fruit production [3]. Pruning can stimulate new growth development, promote nutrient and water transport, and improve canopy structures to enhance air circulation and sunlight penetration. On the other hand, training involves purposefully guiding the growth of branches and stems to achieve specific shapes or structures, such as espaliers or topiaries [4]. In fruit trees, secondary xylem is responsible for transporting water and minerals from the roots to the leaves and fruit [5], and xylogenesis, especially spatiotemporal xylogenesis, is implicated in certain aspects of fruit development [6, 7]. In ornamental horticulture, where aesthetics take precedence [8], wood formation plays critical roles in the formation of flowering patterns [9], and ornamental features like statures, canopies, barks and foliage, contributing to overall visual appeal of plants [10]. To optimize fruit production, the balance between vegetative growth (stem and leaf development) and reproductive growth (flower and fruit development) is meticulously monitored and adjusted. Notably, the wood formation characteristics of the rootstock can also affect the overall growth and performance of the grafted plants [11]. Addressing these challenges necessitates a comprehensive understanding of the molecular mechanisms governing the regulation of wood formation. Such insights may open up new avenues for genetic engineering in horticulture plants and forest trees, offering opportunities to enhance a diverse range of horticultural traits and elevate wood productivity in forestry.

Wood formation is a complex and continuous developmental process encompassing at least five processes [12–14]: (1) cell division originating from vascular cambium [15, 16]; (2) cell expansion [17]; (3) biosynthesis and deposition of secondary cell wall (SCW) [18, 19]; (4) programmed cell death (PCD) [20]; (5) heartwood formation [21]. At maturity, wood is mainly composed of the remains of SCWs comprising cellulose (40–80%), hemicellulose (10–40%), and lignin (5–25%) [22], and its quality is largely determined by the proportions of these SCW compositions of SCWs [23]. Consequently, it holds immense significance to unravel the molecular regulatory mechanisms governing the third process of wood formation, namely, SCW biosynthesis and deposition.

Present knowledge has shown that cell wall (including SCW) formation involves at least 2000 genes in Arabidopsis [24], including 54 structural genes with known or putative functions and a large number of regulatory genes, which participate in the biosynthesis and assembly of cell walls [25]. The expression of these genes undergoes modulation by developmental rhythms and various environmental cues through a large complex regulatory network involving phytohormones [26, 27], transcription factors (TFs) [28–30], epigenetic regulation [31, 32], and post-transcriptional regulation [33–35]. Current knowledge has shown that the vast majority of genes in eukaryotes, including those implicated in SCW formation [36], are principally regulated at the transcriptional level [37, 38]. The breakthroughs in understanding the regulatory networks of SCW formation over the last two to three decades have been significantly promoted by advancements in transgenic approaches and high-throughput sequencing data analysis. At present, a total of 517 TFs of 58 gene families are known to play various regulation roles in the processes of wood formation in poplar [39].

There are several reviews that cover multiple processes of wood formation as aforementioned [14, 18, 40–43]. Simultaneously, a dozen of reviews have specifically delved into particular aspects of the transcription regulation of SCW formation [31, 44–51]. Despite these efforts, the increasing volume of data and results underscores the existing gap in a systematic review on SCW transcription regulation that encompasses a broader range of regulatory genes [52–55], regulatory relationships [56–58], and network motifs (e.g., feed-forward loops) [57, 59, 60]. Furthermore, there is a pressing need to address the specific differences in the transcriptional regulation of SCW formation between herbaceous and woody plants, given that the knowledge derived from different species often befuddles tree biologists [61, 62]. Finally, the various modifications, such as epigenetic [63–65], posttranscriptional [66, 67], and post-translational modifications [68–71] that do not change the DNA-sequence but affect the expression and functions of the genes involved in SCW formation should be specially addressed.

Though many horticultural practices involve the modulation of wood formation processes to enhance horticultural traits, studies on the molecular mechanisms of the SCW formation have been exclusively conducted in two model plant species: Arabidopsis and Populus. This review mainly focuses on the recent advancements in the aforementioned aspects of SCW transcriptional regulation in vessel and fiber cells of *Arabidopsis thaliana* [72, 73] and *Populus trichocarpa* (poplar) [74], with the evidence from other woody plants including other poplar tree species and *Eucalyptus* occasionally cited*.* Due to the conservation of regulatory mechanisms under SCW formation, we hope that this review could promote SCW studies in other horticultural and forest species.

## Transcriptional regulation of SCW formation

### Hierarchical gene regulatory network (HGRN) in herbaceous plant Arabidopsis

In Arabidopsis, significant metabolic commitment to SCW deposition typically occurs during the maturation of vessels and fiber cells in hypocotyls and developing inflorescence stems [75, 76]. Despite its herbaceous nature, Arabidopsis has been used as an excellent model plant for uncovering the molecular mechanisms underlying secondary growth regulation and SCW biosynthesis [72, 73, 77]. Many genes, particularly TFs and SCW biosynthetic genes, as well as their regulatory relationships have been identified owing to their pivotal roles in SCW formation and wood property determination [20, 56, 71, 77–88].

#### The HGRN of SCW formation in Arabidopsis

As the evidence accumulates in the last two to three decades, a conserved pyramid-shaped HGRN consisting of four hierarchical transcription regulation levels has been emerged and is considered to primarily control SCW formation in vessel and fiber cells of Arabidopsis [18, 19, 42, 46, 87, 89].

**Figure 1:**
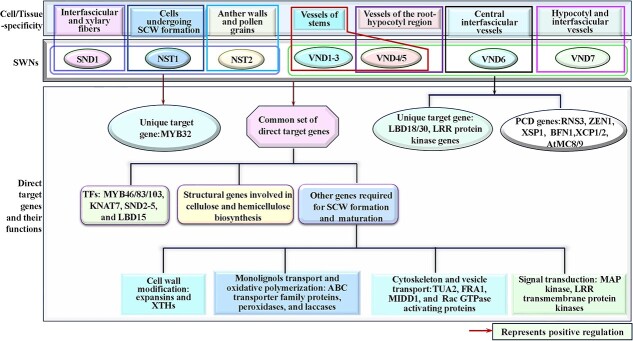
The expression patterns and direct target genes of secondary cell wall NAC TFs (SWNs) in Arabidopsis. The top panel shows the cell/tissue-selectivity of different SWNs of Arabidopsis, while the bottom panel shows the direct target genes and their functions of SWNs in the SCW formation.

The first-level TFs , referred to as SECONDARY WALL NACs (*SWNs*), in the HGRN, which include VASCULAR-RELATED NAC DOMAIN1 (*VND1–7*) [78, 86, 90] and NAC SECONDARY WALL THICKNING PROMOTING FACTOR1 (*NST1–3*) [85, 91] (Figure 1), are generally considered as ‘master switches’, and can pass ‘the commands of SCW formation initiation’ to downstream genes by binding to the 19-bp secondary wall NAC-binding elements (SNBEs) in their promoters with differential binding affinities [44, 92–94]. Among these *SWNs*, the high redundancy of *VNDs* in vessels may signify the importance of vessels in plant survival because SCW defects in vessels are detrimental to plant growth [95]. It is noteworthy that although *SWNs* are expressed in different cell types, *SND1* (also named as *NST3*) is specifically in interfascicular and xylary fibers [96]. *NST1* is found in various tissues undergoing SCW formation; *NST2* is present in anther walls and pollen grains [97]; *VND1–5* are located in vessels of stems; *VND4/5* are specifically in vessels in the secondary xylem of the root-hypocotyl region [86]; *VND6* is identified in the central interfascicular vessels; and *VND7* is observed in the hypocotyl and interfascicular vessels [98]. Despite this cell-specific expression, they share the ability to activate a common set of direct target genes. These include *MYB46/83/103*, *KNAT7, SND2–5, LBD15*, cellulose and hemicellulose biosynthetic genes, and other genes required for SCW formation and maturation, such as PCD, cell wall modification, cytoskeleton and vesicle transport, signal transduction, and monolignols transport and oxidative polymerization [85, 87, 88, 90, 92, 94, 99] (Figure 1). It is also noteworthy that SWNs rarely, if ever, directly regulate monolignol biosynthetic genes [50, 92]. In addition, SWNs also regulate some unique target genes. For instance, VND7 regulates *LBD18/30* and LRR protein kinase genes [100–102], and SND1 exclusively regulates *MYB32* [45] (Figure 1). These findings revealed that although *SWNs* are functionally interchangeable in activating SCW formation, they have evolved to possess distinct regulatory roles for the different cell types [50, 85]. For example, although SND1 and NST1/2 have the ability to activate genes involved in PCD as vessel-specific VND1–7 do [20], they cannot activate PCD because there are other unknown factors that dictate the turning-on of PCD process in fiber cells [92]. Moreover, the same SWNs can also exert different regulation to different target genes. For example, SND1 shows a much lower activation strength toward the SNBE1 sites in *MYB103* promoter than in *MYB46* promoter, which leads SND1 to activate *MYB46* stronger than it does to *MYB103* [92]. These unique and differential regulation of SWNs can be ascribed to the variant SNBE sites in the promoters of downstream genes [92, 94].

**Figure 2:**
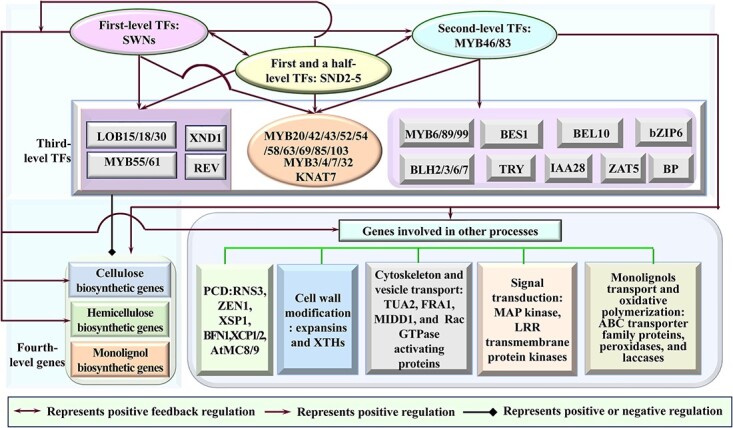
The hierarchical gene regulatory network (HGRN) controlling secondary cell wall (SCW) formation in Arabidopsis. The first-level transcription factors (TFs), SWNs, include SND1, NST1/2, and VND1–7; first and a half-level TFs include SND2–5; the second-level master switches includes MBY46/83. Only genes that have been proven to be involved in regulation of SCW biosynthesis are included in the diagram.

The second-level TFs, *MYB46/83*, which are directly activated by fist-level SWNs through binding to SNBEs with differential affinities [79, 81, 103], function redundantly as master switches. They also serve as converging points in the HGRN for SCW formation of fibers and vessels [49] (Figure 2). MYB46/83 directly activate not only the TFs in the lower-levels of the HGRN, such as the third-level TFs, including *KNAT7*, *MYBs*, and *AtC3H14* [104, 105], but also the fourth-level structural genes involved in SCW-specific cellulose, hemicellulose, and monolignol biosynthesis [49, 81, 103], through binding to 7 bp secondary wall MYB-responsive elements (SMRE) and/or 8p MYB46-responsive *cis*-regulatory elements(M46RE) in their promoters [105, 106] (Figure 2). It is interesting that among eight xylan biosynthetic genes, MYB46 directly regulates *FRA8*, *IRX8*, *IRX9*, and *IRX14* by binding to M46RE motifs in their promoters, but it does not directly regulate *PARVUS*, *IRX10*, *IRX15*, and *IRX15-L* due to the lack of M46RE motifs in their promoters [49]. MYB46/83 also directly activate *BEL10*, *bZIP6*, *TRY*, *IAA28*, *BLH2/3/6*, and *ZAT5* [105], which are preferentially expressed in xylem tissues [87] (Figure 2). In addition, like first-level SWNs, MYB46/83 also directly activate the genes involved in PCD, cell wall modification, cytoskeleton and vesicle transport, signal transduction, and monolignol transport and oxidative polymerization processes, all closely linked to SCW formation [103, 105] (Figure 2). Recently, it has been reported that *MYB46/83* are also directly activated by SND2/3/4/5, which are distinctly expressed in interfascicular fibers and xylem, and can be activated by different SWNs [88]. Notably, overexpression of *SND2/3/4/5*-VP16 induces the expression of the same set of downstream genes including *MYB46/83* as SWNs do by binding to SNBEs in their promoters, suggesting that they are positioned between the first-level *SWNs* and second-level *MYB46/83* in the HGRN [88] (Figure 2).

As the third-level TFs, *MYB3/4/7/32/20/*42/*43/52/54/58//63/69/79/85/103* and *KNAT7* are directly activated by SWNs [45, 87, 92, 107], SND2–5 [88], and MYB46/83 [81, 104, 105]. *LOB15/18/30* [100, 101], *MYB55/61* [108, 109], *REV* [110], and *XND1* [57] are directly activated only by SWNs but not by MYB46/83 [46], while *MYB6/89/99*, *AtC3H14*, *BES1, BEL10, bZIP6, TRY, IAA28, BLH2/3/6*, *BP*, and *ZAT5* are directly activated only by *MYB46/83* but not by *SWNs* [49, 50, 105] (Figure 2). These third-level TF*s*, together with SWNs and MYB46/83 [90, 111], act as activators or repressors to selectively regulate the expressions of fourth-level structure genes, mainly including cellulose synthase genes (*CesA4/7/8*) [112], xylan biosynthetic genes (*IRX7*/*8/9/10/13/14/15/15 L* and *PARVUS*) [95, 113–115], and monolignol biosynthetic genes (*PAL*, *C4H*, *4CL*, *HCT*, *C3H*, *CCoAOMT*, *F5H* (also named *CAld5H* or *AldOMT*)*, COMT*, *CCR*, *CAD*, and *CSE*) [116, 117], whose proteins catalyze the respective enzymatic reactions in SCW component biosynthesis [29] (Figure 2).

**Figure 3:**
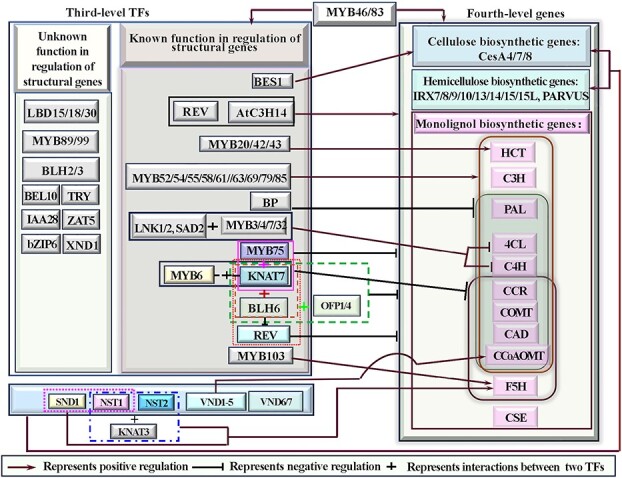
Multi-level regulation of fourth-level structural genes by third-level TFs and some master switch TFs in the hierarchical gene regulatory network (HGRN) that governs secondary cell wall (SCW) formation in Arabidopsis. Only genes that have been proven to be involved in regulation of SCW biosynthesis are included in the diagram.

The recent evidence shows the cellulose and hemicellulose biosynthetic genes are mainly regulated by SWNs [87, 92] and SND2–5 [88] that bind to SBNE and/or tracheary element-regulating cis-elements [20], and by MYB46/83 that bind to SMRE elements [118](Figure 1 and2). It is also of noteworthy that BES1 is the only TF that specifically activates cellulose biosynthetic genes among the third-level TFs in the HGRN via the CANNTG E-box motif [119] (Figure 3), and AtC3H14 may function as the other master switch like MYB46/83, which directly activates not only entire SCW biosynthetic genes but also the same-level TFs, such as *MYB52/54/63* and *KNAT7* [104] (Figure 3 and4). In contrast, the monolignol biosynthetic genes are mainly regulated by MYB46/83 and third-level TFs (Figure 3), but to a less degree, by SWNs (Figure 1 and 2). For example, MYB46 has been proven to activate *PAL*, *C4H*, *4CL*, *HCT*, *C3H*, *F5H*, *CCR*, *CAD*, *CCoAOM*, and *CSE* directly via binding to the variants of SMREs that are identical to AC elements [103, 120], which are also known as C1-motif, PAL-box, or H-box, and play a role in coordinating expression of monolignol biosynthetic genes [120]. Among third-level TFs, MBY52/54/55/58/61/63/69/79/85 specifically activate *PAL*, *4CL*, *HCT*, *C3H*, *CCoAOMT*, *CCR*, and *CAD* through binding to the conserved AC elements in their promoters [28, 84, 109, 121, 122], and *C4H* and *COMT* through the degenerated AC elements [123] (Figure 3), while MYB20/42/43 directly activate *HCT* [122] (Figure 3)*. F5H,* the only monolignol biosynthetic gene without AC elements, is directly regulated by NST1, SND1, and MYB46 [124], and MYB103 [125] as well as heterodimers of KNAT3 and NST1/2 [126] (Figure 3). It is interesting that among SWNs, VND1–5 have been reported to directly activate only one of 11 monolignol biosynthetic genes, *CCoAOMT* [86] (Figure 3). It is notable that MYB3/4/7/32/6/75, KNAT7, and BP, are only currently known repressors in the HGRN, among which MYB3 and MYB4/7/32 only repress *4CL* and *C4H* via interacting with other TFs [127, 128] (Figure 3), while MYB75 negatively regulates the entire SCW formation biosynthetic genes via interaction with KNAT7 [83] (Figure 3). BP directly represses *CCoAOMT*, *COMT, PAL*, *C4H*, *4CL*, and *CAD* by binding to BP-binding sites and/or KN-1 motifs in their promoters [129] (Figure 3). Notably, the current HGRN is still incomplete because of new TFs, regulatory relationships as well as network motifs are still being revealed [110, 111].

**Figure 4:**
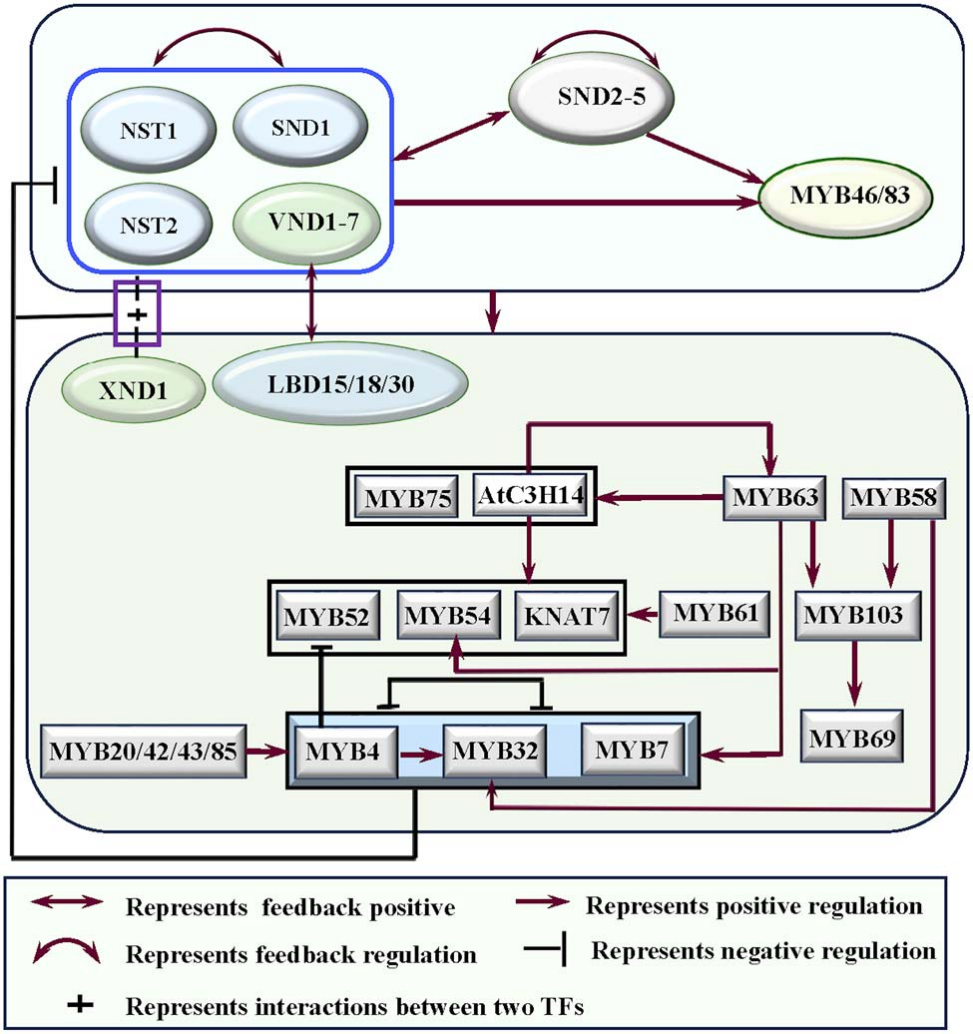
Complex regulatory relationships are represented by various network motifs among transcription factors (TFs) in the hierarchical gene regulatory network (HGRN) of Arabidopsis.

#### Characteristics of the HGRN in Arabidopsis

It is generally thought that the HGRN employs multiple feed-forward loops (FFLs) to control SCW formation, where one TF in an upper-level regulates a *TF* in the next-level and then they together regulate their common downstream targets [50]. However, as more facts are being revealed, the complexity of regulatory relationships in the HGRN has gone beyond our early imagination, which appears to involve complex and wrapped transcriptional regulation relationships [110, 111].

The HGRN for SCW formation is ultimately composed of multiple network motifs, which are discrete patterns of regulation that occur more frequently than expected from randomized networks [130]. As described earlier [131], the typical network motifs contain three genes (for example, X, Y, and Z) with transcriptional regulation relationships, which can constitute four coherent feedforward loops (C-FFLs) and four incoherent feedforward loops (I-FFLs). For instance, the type 1 C-FFL motif, X → Y → Z and X → Z, where → represents activation [131]. In the HGRN of SCW formation, the third-level *MYB3/4/7/32/20/42/43/52/54/58/63/69/85* and *KNAT7* (Z) are directly activated by the second-level MYB46/83 (Y) [104, 105] and first-level SWNs (X) [132] (Figure 2), forming multiple C-FFLs. Additionally, there also exist many C1-FFLs among *SWNs* (X), *SND2–5* (Y), and *MYB46/83* (Z) [88] (as shown in Figure 2). *SWNs* (X), *MYB46/83/AtC3H14* (Y), and *MYB52/54/63/KNAT7* (Z) [104] (Figure 4); *MYB46/83* (X), *MYB20/42/43/63/*85 (Y), and *MYB4/7/32* (Z) [104, 105] (Figure 4). Meanwhile, the HGRN also includes many I1-FFLs, which represent $X\to Y\dashv Z$ and $X\to Z$ (where $\dashv$ indicates a repression) [131]. For example, *MYB46/83* (X), *MYB4/7/32* (Y), and *C4H* (Z) [104, 133]; *MYB46/83* (X), *KNAT7* (Y), and SCW biosynthetic genes (Z) [55, 104, 134–136] (Figure 3); *SWNs* (X), *KNAT7* (Y), and cellulose and hemicellulose biosynthetic genes (Z) [55, 134–136] (Figures 3 and4). It is known that the C1-FFL is a ‘sign-sensitive delay’ element and a persistence detector, while the I1-FFL is a pulse generator and response accelerator [131]. Compared with I1-FFLs, there are more C1-FFLs in the HGRN of SCW formation. However, we still do not have a full understanding of the roles played by C1-FFL and I1-FFL in the HGRN of SCW formation. In addition, *MYB4/7/32*, which are activated by MYB46/83/58/63 directly [104] (Figure 4), can repress SND1, NST1/2, and VND6/7 [107] (Figure 4), and their own transcription [104], forming multiple negative feed-back loops (FBLs) and negative autoregulation. *XND1*, directly activated by SWNs, can repress *VND6* in a negative FBL [57] (Figure 4). On the contrary, positive FBLs also present in the HGRN of SCW formation universally (Figure 2-4). For example, *SND2/3/4/5*, which can be activated by SWNs, upregulate not only themselves but also *SWNs* in a positive autoregulation and/or a FBL respectively (Figure 4), which is evidenced by the fact that overexpression of *SND2/3/4/5*-VP16 activate themselves and several *SWNs* [88]. LBD15/18/30, as the direct targets of VND6/7, regulate the expression of *VND6/7* in positive FBLs [92, 101, 102]. In addition, SND1 and VND7 also have positive autoregulation abilities [102, 107]. Among these FBLs, the negative autoregulation of TFs, like MYB4/7/32 [104], can potentially speed up the response time of gene circuits when a TF has a strong promoter, and reduces cell–cell variation in protein levels, whilst the positive autoregulation has been reported to enhance the sensitivity to signals, and generate a switch-like response [137, 138]. Moreover, it was shown that more robust networks tend to have larger numbers of positive FBLs and smaller numbers of negative FBLs [139], which is also consistent with that there are more positive FBLs than negative FBLs in the HGRN of SCW formation as described in Figure 2 to4. Altogether, inclusion of various types of FFLs and FBLs as well as positive and negative autoregulation is a prominent feature of developmental network that tends to act slowly and can irreversibly trigger a transient developmental instruction [131], which can render dynamic and adaptative aspects of SCW formation [101, 140].

Third-level TFs and fourth-level structural genes in the HGRN are subject to complex and intricate transcriptional regulation from multiple upper-level TFs (Figure 2 and3). For instance, *MYB20/42/43/52/54/58/61/63/69/79/85, KNAT7,* and *AtC3H14* are directly activated by MYB46/83 [49, 103, 141], SND2–5 [88], and SWNs [20, 85], forming many FFLs (Figure 2 and3). The fourth-level structural genes are synergistically regulated by SWNs [86], SND2/3/4/5 [88], MYB46/83 [105, 106], and third-level TFs, such as AtC3H14 [121], KNAT7 [55, 126, 142], and MYB20/42/43/85/58/63 [84, 122] (Figure 2 and3). In addition, there are some intra-level regulatory relationships among the third-level TFs (Figure 4). For instance, MYB63 positively regulates the transcription of *MYB4/7/32/54/75* and *AtC3H14*; AtC3H14 strongly activates the transcription of *MYB52/54/63* and *KNAT7,* MYB61 activates *KNAT7,* MYB63/58 directly activate *MYB103* that in turn positively regulates *MYB69*, while MYB4/7/32 negatively regulate expression of *MYB52* [104]. MYB20/42/43/85/63 can activate the expression of *MYB4/7/32* [122]. Given more upper-level and intra-level of regulatory above, the third-level TFs and fourth-level structural genes are subjected to more sophisticated and intricate regulation, involving more network motifs. This complexity allows Arabidopsis SCW formation to better cope with developmental rhythm and ever-changing environments.

According to the Hussey et al's study [45], the master switches at the upper levels of the HGRN may directly regulate some structural genes at the four-level, with a preference to them over the TFs located at lower hierarchical levels. This direct regulation of higher-level TFs over structural genes at lower levels provide a rapid regulation of specific SCW component formation, specifically for SWNs (Figure 2), which is very useful when plants are under adverse environmental condition [143]. In addition, this kind of transcriptional regulation structure may pass down some transient or weak activation signals from the master switches directly to the bottom-layered biosynthetic genes without activating the middle-level regulatory layers (Figure 2), avoiding employment of the whole HGRN to response [144], which is useful for plants to make decision on SCW formation on fluctuating external signals. The regulatory commands that are relayed to multiple successive layers and ultimately to the structural genes at the bottom of the network, may activate a large number of targets whose proteins are needed for synthesis many SCW components.

Due to whole genome duplications, many genes in the HGRN function redundantly. Consequently, a single mutant in one gene generally does not show a phenotypic alternation of SCW [81, 145]. For instance, the knockout of *SND1* has no alternation of SCW thickness, whereas simultaneous inhibition of *SND1* and *NST1* leads to loss of entire SCW formation in fibers of stems [145]. Simultaneous RNAi inhibition of both *MYB46* and *MYB83* results in a reduction in SCW thickening in fibers and vessels, and double knockout of *MYB46* and *MYB83* causes a lack of SCW in the vessels, whereas knockout of either *MYB46* or *MYB83* has no discernable effects on SCW deposition in fibers or vessels [81]. Additionally, although *MYB52/54/69/103* are common downstream TFs of SND1, NST1/2, and VND6/7, they regulate SCW formation only in interfascicular and xylary fiber cells, but do not impact the SCW formation in the vessels (Zhong, Lee et al. 2008), which necessitates more specifically designed experimental system to elucidate their tissue-specific regulation. It is obvious that vascular plants have evolved the mechanisms that incorporate functional redundant genes and regulatory relationships to safeguard the SCW formation that is essential for the survival of vascular plants.

**Figure 5:**
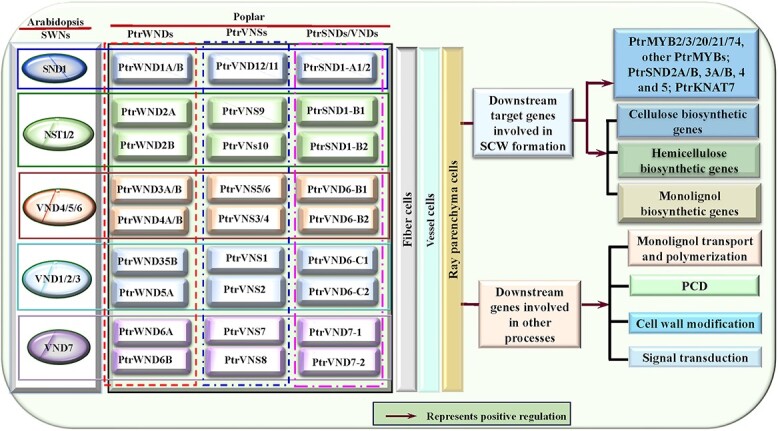
Illustrates the conversion between Arabidopsis Secondary Wall NACs (SWNs) and their poplar counterparts, referred to as Wood-Associated NAC Domain proteins (WNDs) or those with alternative names such as VNs, VNSs, or VNDs used in existing literature. It also highlights the primary functions of their target genes in Secondary Cell Wall (SCW) formation in poplar.

Compared with transcriptional activators, there are only a few transcriptional repressors in the HGRNs for SCW formation that have been recognized, such as *MYB3/4/7/32/6/75* [127, 128, 146], *KNAT7* [135], and *BP* [129], most of which are evidenced to repress the monolignol biosynthesis. These repressors are essential for attenuating and patterning of the expression of genes in the HGRNs and adapting to ever-changing environmental condition.

### The HGRN in poplar, a model woody plant species

After the release of *P. trichocarpa* genome [74], it emerged as a model tree species for investigating various challenges specific to perennial woody plants, including secondary growth, long-term perennial growth, and seasonality (e.g., dormancy and bud break). These issues are not as easily addressed with the herbaceous Arabidopsis [147–149]. Meanwhile, due to the evolutionary conservation of transcriptional regulation of SCW biosynthesis [46, 132, 150], most knowledge of the HGRN for SCW formation gained from Arabidopsis can be broadly applied to other species, such as poplar. However, there are also distinctions in the HGRN of poplar when compared to that of Arabidopsis.

**Figure 6:**
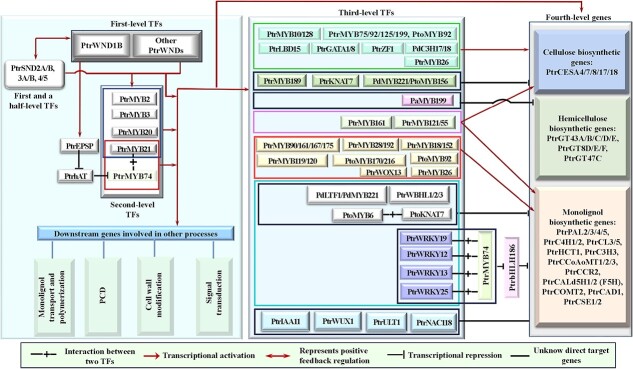
The multi-layered HGRN controlling secondary cell wall (SCW) formation in Poplar. Only genes that have been proven to be involved in regulation of SCW biosynthesis are included in the diagram.

It has been shown that poplar employs six pairs of SWN homologs as first-level master switches, including 12 wood associated NAC domain TFs (*PtrWND1A/B–6A/B*) [62], which are also referred to as *PtrVNSs* [151] or *PtrSNDs*/*PtrVNDs* in other studies [152]. For more detailed understanding of gene conversion and functions, please consult Figure 5. PtrWNDs, the counterparts of SWNs in Arabidopsis, directly activate some downstream TFs and structural genes involved in SCW formation as well as other SCW-associated processes, such as PCD, cell wall modification, monolignols transport and oxidative polymerization, in across multiple developing fiber, vessel, and ray parenchyma cells through binding to SNBEs in their promoters with different strengths [62, 89] (Figure 5). For example, even though all *PtrWNDs* are able to complement the SCW defects in the *snd1 & nst1* double mutant, only *PtrWND2B/6B* have sufficient strength to drive ectopic SCW deposition in parenchyma cells when they are overexpressed [62]. The PtrWND*6*A/B can sufficiently drive genes encoding xylem cysteine peptidase 1, polygalacturonase, and peroxidase, whereas PtrWND2A/B effectively activate the *PtrMYBs* and *PtrCesAs* [151]. Additionally, some PtrWNDs can autoregulate their own gene expression as their counterparts in Arabidopsis do [89]. It is notable that *PtrWNDs* show a large difference in regulatory strengths between homologous gene pairs in some cases. For example, PtrWND2A effectively activates the *PtCesA18* promoter but does not exert significant regulation on *PtCesA17* promoter, while PtrWND2B shows opposite regulatory strengths on these two target gens [151]. Furthermore, PtrWNDs can form homodimers or heterodimers with different transcriptional activities [62, 153], contributing to the fine regulation of their downstream genes for SCW formation in different tissues or cells and in response to different environmental cues. Altogether, upon receiving internal and external signals, *PtrWNDs* act as the start point and pass the ‘commands of SCW formation initiation’ via different regulation pathways with different regulatory strengths in the HGRN, leading to SCW formation in developing xylem of poplar.

The second-level TFs, *PtrMYB2/3/20/21/74*, directly activated by PtrWNDs, function as the master switches as *MYB46/83* do in the HGRN of Arabidopsis [62, 89] (Figure 6). Unlike MYB46/83, PtrMYB2/3/20/21 exhibit marked differences in activating their target genes. These differences arise from their distinct expression in different organs and tissues and/or by their differential binding affinities to the SMREs in the promoters of their target genes [154, 155]. Notably, PtrMYB74 (also known as PtrMYB50), which lacks a functional counterpart in Arabidopsis, shares a substantial number of target genes with PtrMYB21 [60]. Therefore, *PtrMYB74* is suggested to be an additional second-level master switch in the HGRN of poplar (Figure 6). It is important to highlight that PtrMYB21 and PtrMYB74 were found to form a heterodimer to regulate downstream genes more efficiently [60] (Figure 6). Moreover, *PtrEPSP*, encoding 5-enolpyruvylshikimate 3-phosphate synthase with a helix-turn-helix motif and directly activated by PtrWND1B, can repress the expression of the transposase family gene*, PtrhAT.* PtrhAT, in turn, serves as the direct upstream negative regulator of *PtrMYB21,* therefore positively regulating SCW biosynthetic genes [156] (Figure 6). Furthermore, *PtrSND2A/B, 3A/B, 4/5* (corresponding to *PtrNAC154/156, 105/157, 150/151,* respectively), like their Arabidopsis counterparts, *SND2–5*, are directly activated by PtrWNDs. These transcription factors positively regulate not only *PtrMYB2/3/20/21* but also *PtrWNDs* through binding to SNBEs in their promoter [88] (Figure 6).

The third-level TFs, including *PtrMYB10/128* (*MYB103* in *A. thaliana* (*At*))*, PtrMYB26/31/158/189* (*MYB69* in *At*)*, PtrMYB90/167/161/175* (*MYB52/MYB54* in *At*), *PtrMYB75/92/125/199* (*MYB42/MYB85* in *At*)*, PtrNAC118* (*XND1* in *At*)*, PtrZF*1, *PtrGATA1/8*, *PtrKNAT7*, *PtrLBD15*, *PtrIAA11*, *PtrWUS1*, *PtrWOX13*, and *PtrULT1* [89]*, PtrMYB28/192* (*MYB58/MYB63* in *At*) [120]*, PtrMYB119/120 (MYB75* or *PAP1* in *At)* [157, 158]*, PtrMYB55/121* [89, 159] and *PtoMYB170/216* (*MYB61* in *At*) [160, 161]*, PtrWRKY12/13/25/19* [89, 162], *PtrWBLH1/2/3* (*BLH2/3/6a* in *At*) [60, 89], *PtrMYB18/152* (*MYB20/43* in *At*) [120, 163], *PtrMYB6/126 (MYB5* in *At*) [164], *PdC3H17/18 (AtC3H14* in *At*) [165]*, PaMYB199* (*MYB20/42/85* in *At*) [58]*, PtoMYB156* [166]*, PdMYB221* [167] or named as *PdLTF1* [168] (*MYB4* in *At*), and *PtoMYB92* (*MYB42/85* in *At*) [169]*,* directly activated by first-level TFs, second-level TFs, and/or first and a half-level *PtrSND2A/B, 3A/B,* and *4/5*, selectively regulate fourth-level structural genes. These include cellulose biosynthetic genes (*PtrCESA4/7/8/17/18*) [170, 171], hemicellulose biosynthetic genes (*PtrGT43A/B/C/D/E*, *PtrGT8D/E/F*, and *PtrGT47C*) [172–174], and monolignol biosynthetic genes (*PtrPAL1/2/3/4/5*, *PtrC4H1/2*, *PtrCL3/5*, *PtrHCT1*, *PtrC3H3*, *PtrCCoAoMT1/2/3*, *PtrCCR2*, *PtrCALd5H1/2*, *PtrCOMT2*, *PtrCAD1, and PtrCSE1/2*) [175, 176] (Figure 6). For instance, PtrMYB10/128, PtrMYB75/92/125/199, PtrMYB150, PtrLBD15, PtrZF1, PtrGATA1/8, and PdC3H17/18 activate promoters of entire or several SCW biosynthetic genes [89, 165], whereas PtrMYB189 [177], PtrXND1 [178], PtrKNAT7 [134]*,* PdMYB221 [167] and PtoMYB156 [166]*,* PtoMYB6 [164]*,* PaMYB199 [58] selectively repress SCW biosynthetic genes (Figure 6). PtrMYB161 [59] and PtrMYB121/55 [159] can activate cellulose and monolignol biosynthetic genes (Figure 6). Notably, PtrMYB161 regulates the *PtrWND1A/B* and *PtrWND2A/B* in a negative FFL [59]. Additionally, PtrMYB18/152 [163], PtrMYB26, PtrMYB28/192*,* Ptr90/161/167/175 [120]*,* PtrMYB119/120 [157], Pto170/MYB216 [160, 161]*,* and PtoMYB92 [169] selectively activate all or several monolignol biosynthetic pathway genes, whereas PtoMYB6 (via interaction with PtoKNAT7) [164], PtrWRKY12/13/19/25 [162], PtrWBHL1/2/3 [179], and PtrWOX13 [54] selectively repress all or several monolignol biosynthetic pathway genes (Figure 6). It is also worth noting that as the poplar orthologs of Arabidopsis MYB4*,* PdMYB221 [167] and PtoMYB156 [166] directly represses multiple SCW biosynthetic genes, such as *PdCESA7/8*, *PdGT47C*, *PdCOMT2*, and *PdCCR1*, and *PtrCESA17, PtrC4H2* and *PtrGT43B,* based on the transcriptional activation assays, which is inconsistent with the conclusion that PdLFT1 only represses *4CL* via directly binding to its promoter in its unphosphorylated state [168, 180]. Additionally, PtrMYB26 is reported to activate monolignol biosynthetic genes [89], whereas its homology, PtrMYB189 negatively regulates entire SCW biosynthesis genes [177]. These studies suggests that the functions of some *PtrMYB* homologs involved in regulation of SCW formation have diverged. These pieces of evidence in poplar again indicate that the fourth-level structural genes are regulated by the sophisticated and intertwined transcriptional regulation relationships comprising at least three upper-level TFs, and that though more genes are involved in SCW formation in poplar than Arabidopsis, the backbone of the two HGRNs still resemble to each other.

It should be noted that novel TFs or non-TFs with regulatory functions involved in SCW formation have continuously been identified in the last few years, such as *PdIQD10* [181], *PtrMYB120* [157], *PtrGATA12* [52]*, PtrHAT22* [53], *PtrGATA12A* [54], *PtrHB3* and *PttHB4* [182], *PtrAP17/45* [183]*, PtrFLA40/45* [184]*, PnMYB2* [185], *PagERF81* [186]. However, we do not know the exact positions of these genes in the poplar HGRN aforementioned. These newly identified genes involved in SCW further demonstrate that the HGRNs for SCW formation are not fully identified and hidden nodes need to be identified to understand the functions of the HGRN in the future.

### The differences of the HGRNs between the Arabidopsis and poplar

Compared with the annual herbaceous Arabidopsis, the perennial woody poplar not only necessitates a massive SCW formation but also requires more heterogeneous SCWs to support huge bodies, facilitate transport water/nutrients over long distance and adapt to seasonal changes and various environmental stresses [14]. Consequently, SCWs of poplar show diverse characteristics across various tissues, markedly differing from those in herbaceous Arabidopsis in terms of SCW structure and chemical composition [147, 187, 188]. Correspondingly, some variations in the HGRNs between these two species have evolved to guarantee the generation of the essential and divergent SCW components. The major differences of the HGRNs of two species can be summarized as following:

First, some ortholog genes in the two HGRNs exhibit differentiation in both expression levels and functionality between the poplar and Arabidopsis. For instance, mRNAs of all expressed *PtrWNDs* are ubiquitously accumulated in all three types of cells$,$vessels, fibers and ray parenchyma cells, in the developing xylem of poplar [62], where they positively regulate the genes involved in SCW biosynthesis, PCD, cell wall modification, and monolignol polymerization and transport, and signal transduction [89] (Figure 5). In Arabidopsis, *SWNs* are primarily expressed in vessels and fibers of inflorescence stems and mature hypocotyls with obvious functional differentiation [151] (Figure 1). For example, SND1 and NST1/2 are responsible for activating the genes involved in SCW biosynthesis, cell wall modification, and monolignol polymerization and transport in fiber cells [92] (Figure 1), while VND1–7 activate genes involved in the same processes as SND1 and NST1/2 do, plus PCD in vessel cells of inflorescence stems and/or mature hypocotyls [20, 86] (Figure 1). In addition, each of *SWNs* only produces one form of the transcript in Arabidopsis as well as transgenic poplar overexpressing *SWNs*, whereas their poplar homologs, *PtrWND1B*, *PtrWND3A/B*, and *PtrWND5A,* undergoes alternative splicing (AS), among which *PtrWND1B* has intron-retained (IR) splice variant not only in poplar but also in the *PtrWND1B*-overexpressing Arabidopsis [67, 152]. *MYB69,* activated by NST1 but not by SND1, is a transcriptional activator that positively regulates lignin biosynthesis of Arabidopsis [84, 87], whereas its counterpart in poplar, *PtrMYB189* (also namely *PtrMYB158/31/26*), activated by PtrWNDs [89], acts as a repressor of SCW biosynthesis through directly binding to the promoters of *PtrC4H2*, *PtrCOMT2*, *PtrGT43B*, and *PtrCesA2B* [177]. MYB85 in Arabidopsis only positively regulate phenylalanine and monolignol biosynthesis [122], whereas its counterparts in poplar, *PtoMYB92*, not only promote the accumulation of lignin but also inhibit the synthesis of hemicellulose [169]. MYB103 specifically regulates *F5H* expression in the Arabidopsis inflorescence stem [125], whereas its counterparts in poplar, PdMYB10/128 (or PtrMYB10/128)*,* activate the genes involved in the biosynthesis of three SCW components [189].

Second, poplar and Arabidopsis may have evolved their unique regulatory cascade in the HGRNs of SCW formation. For example, although PtrWND1B (PtrSND1-B1) in poplar and SND1 in Arabidopsis directly activate 10 and 14 TFs respectively*,* they share only one common target, namely, *PtrMYB21* in poplar and its counterpart *MYB46* in Arabidopsis, manifesting a significant divergence in the two HGRNs [60]. Additionally, downstream of such a NAC-MYB regulatory chains in the HGRNs, the targets of MYB hubs become distinctly different in two species [190]. For instance, PtrMYB21 directly activates eight SCW biosynthetic genes and 10 TFs, and its counterparts in Arabidopsis, MYB46, directly regulates 12 SCW biosynthetic genes and 17 TFs in the HGRNs of SCW formation. Among the SCW biosynthetic genes and TFs regulated by PtrMYB21 and MYB46, only six target genes, including two structural genes, *PAL1* vs *PtrPAL2*, and *IRX14-L* vs *PtrIRX14-L*, and four TFs, *MYB52* vs *PtrMYB90/161/175*, *BLH2/3/6* vs *PtrWBLH1/2/3*, are common [60]. Moreover, *PtrMYB152*, an ortholog of Arabidopsis *MYB43* activated by SWNs, is regulated by PtrWNDs, except PtrWND2B, implying the presence of a *PtrWND2B*-independent regulation pathway that governs SCW biosynthesis [89].

Third, the HGRN encompasses more genes in poplar compared to Arabidopsis. Based on the existing literature, the HGRN of Arabidopsis contains at least 53 TFs [85–87], including ten first-level *SWNs*, four first and a half-level TFs (*SND2–5*), two second-level TFs (*MYB46/83*), and 37 third-level TFs (Figure 1-3), while there are at least 70 TFs in the HGRN of poplar [60, 62, 89], including 12 first-level *SWNs*, six first and a half-level TFs (*PtrSND2A/B*, *3A/B*, and *4/5*), five second-level TFs, and 47 third-level TFs (Figure 5 and6). It is important to note that these figures do not represent an exhaustive list of genes in the HGRNs of the two species, and there may be undiscovered genes that have not been documented.

## The HGRNs perceive external signals and respond accordingly to modulate SCW formation

**Figure 7:**
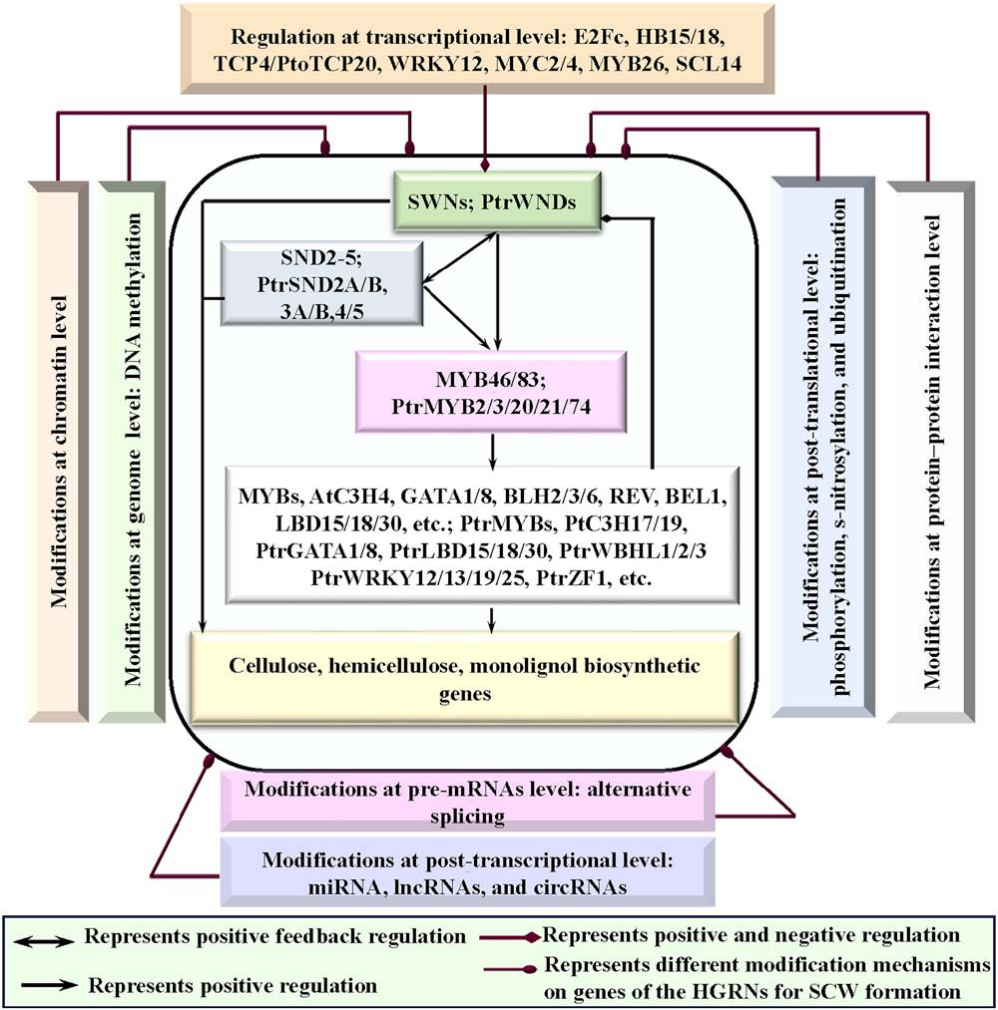
Multi-level modification of hierarchical gene regulatory network (HGRN) modulate the biosynthesis of heterogenous secondary cell wall (SCW).

As sessile organisms, plants are constantly subjected to various environmental stresses and cues. The HGRNs can function as an integral part of the intricate mechanism to respond to external stimuli and adjust SCW formation for enhanced survival and adaptation. It has been reported that certain first-level TFs in the HGRNs are regulated by other TFs, with the latter typically involved in response to various environmental cues [50] (Figure 7). For instance, UV-B increases the expression of *E2Fc* [191], which directly regulates *VND6*/7 in Arabidopsis, and E2Fc can activate or repress *VND7* expression in a dose-dependent manner [110]. HD-ZIP III subfamily genes, including *REV*, *PHB*, *PHV*, *HB15*, and *HB8,* respond to salinity, drought, ABA, and biotic stresses due to the steroidogenic acute regulatory protein-related lipid transfer and MEKHLA domains present in the C-termini of these TFs, associated with various chemical and physical stimuli [192, 193]. Among these TFs, HB15 regulates SCW development by directly inhibiting the expression of *SND1*, *NST2,* and *AtC3H14* [194], while HB8 regulates vessel differentiation by directly promoting the expression of *VND6*/*7* [195]. REV and PHV are positive regulators of the final stages of xylem differentiation through directly binding to the promoter of *VND7* [196]. Arabidopsis WRKY12 represses lignin biosynthesis by directly inhibiting expression of *NST2* and *PtrWND2A/B* in the pith parenchyma cells of inflorescence stems [162, 197]. The expression of *WRKY12* is inhibited by Cd stress [193] and activated by hypoxia [198]. WRKY15, induced by oxidative stress and salt [199], inhibits the expression of *VND7* in the vascular protoxylem of Arabidopsis roots [200]. TCP4, which interacts with auxin, gibberellic acid, and abscisic acid- response pathways in plant growth and development [201], triggers SCW biosynthesis and PCD of vessel cells via activating *VND7* by directly binding its promoter [34]. Correspondingly, PtoTCP20, whose homolog in Arabidopsis responds to fluctuating nitrate supply [202], activates *PtoWND6* expression to promote secondary xylem differentiation [203].

Two basic helix–loop–helix TFs, MYC2/4, respond to the blue light signal through interaction with blue light receptor CYR1, leading to the activation of *NST1* expression by directly binding to its promoter, which, in turn, results in an enhancement of SCW thickening [30]. Additionally, low R:FR ratio under shaded light conditions promotes the interaction between PHYB and PIF1/3/4/5, leading to their degradation [204]. The degradation of PIF4 decreases its interaction with MYC2/4, which augments MYCs' transcriptional activation on the *NST1*, resulting in the increase of SCW formation [205]. MYB26, which is downregulated by auxin [206], plays a role in activating SCW formation in endothecium through directly binding to the promoters of *NST1/2* [207]. SCL14*,* which is a key DELLA gene in the gibberellin signaling pathway, can repress monolignol biosynthesis likely by inhibiting *NST1*-*MYB61* cascade in the HGRN of SCW formation in *P. hopeiensis* [208].

These pieces of evidence indicate that the HGRNs governing SCW formation are constantly subjected to the modulation of ever-changing environmental cues and factors, which primarily signal the first-level TFs via some stress-responsive TFs as mentioned earlier. Subsequently, these first-level TFs, which are affected, regulate the SCW biosynthetic genes via regulatory cascades and chains in the HGRNs. This observation aligns with the recognized roles of SCW in coping with ever-changing environmental stresses during plant growth and development [209]. These findings imply that there are some TFs that perceive environmental stresses or cues and operate above the HGRNs to modulate SCW formation.

## Modification genes in the HGRNs for SCW formation

Present knowledge indicates that while the expression levels of most genes are primarily regulated at transcription level [210], they are also subject to modulation through **p**ost-transcriptional, **p**ost-translational, and **e**pigenetic **m**odifications (PPEMs) [211]. Similarly, many genes within HGRNs are influenced by PPEMs [31, 44]. PPEMs offer a mechanism for rapid and dynamic responses at the appropriate time, and they are generally reversible at a small-time scale [212]. Leveraging PPEMs as a strategic response to environmental changes and internal stimuli allows plants to adjust key biological processes for better adaptation and development, demanding relatively few cellular resources [213].

### Epigenetic modification genes of the HGRNs

Epigenetic modifications, mainly comprising DNA methylation and histone modifications, dynamically modulate gene expression without a change in DNA sequence [214], and produce heritable phenotypic changes during plant growth and developmental processes [215, 216].

#### DNA methylation modification genes of the HGRNs

DNA methylation at the 5′ position of cytosine affects the epigenetic regulation of nuclear gene expression and genome stability and is important to many biological processes such as growth and development as well as response to abiotic stresses [217]. Recently, some studies began to unveil the roles of epigenetic modifications in modulating the expression of wood formation-related genes (Figure 7). For instance, in the primary, transition, secondary stems of poplar, the expression levels of two monolignol biosynthetic genes, *PtrPAL2* (Potri.008G038200) and *PtrC4H1* (Potri.013G157900), increase dramatically during the transition from primary to secondary stems due to the change of DNA methylation sites [63]. In addition, the methylated levels of three regulatory genes, *PtrMYB52* (Potri.008G089700, Potri.012G039400, and Potri.015G033600), which directly activates *PtrCCoAOMT1* [89]*,* exhibit noticeable differences in the three developmental stages of poplar stems [63]. Moreover, *WND1B/2A, MYB43/55/83/88*, *CESA4/7/8,* and *PAL1* have differential methylation levels in the intergenic regions of genome in the SCW formation of juvenile and mature wood in poplar [218]. Furthermore, the DNA methylation of *BpNST1/2* and *BpSND1* promoters inhibits their expression, and thereby reducing lignin content of *Betula platyphylla* under the high temperature compared with the low temperature [219].

#### Histone modification genes of the HGRNs

Histone modifications, acting as epigenetic indicators of chromatin states associated with gene activation or repression, typically occur within the N-terminal tails of histone proteins in forms of methylation, acetylation, phosphorylation, ubiquitination, sumoylation, glycosylation, and ADP ribosylation [220]. These modifications play crucial roles in plant growth and development [221] as well as response to abiotic stresses [222]. Recent studies have provided lights on histone modifications involved in SCW formation (Figure 7). For instance, ARABIDOPSIS HOMOLOG of TRITHORAX1, a H3K4-histone methyltransferase that is also involved in dehydration stress response [223], increases H3K4me3 level at the loci of *SND1* and *NST1*, leading to the activation of their expression and the increase of SCW deposition in inflorescence stems of Arabidopsis [70]. Linker histones play a role in stabilizing chromatin structure. Recent evidence suggests their interactions with various proteins including stress-response proteins, HSP90B and HSPA8 [224, 225], which can modulate chromatin conformation and gene expression at specific loci [226]. For instance, the repression of EgMYB1 on monolignol biosynthetic genes is enhanced by the interaction with the drought-inducible linker histone variant EgH1.3 at early stages of xylem differentiation and also in mature ray and parenchyma cells of *Eucalyptus grandis* [32]. PtrHDT3-A/B1/B2, encoding histone deacetylases and their homologs in Arabidopsis involved in ABA and salt stress response [227], function as a corepressor to modulate the compaction of chromatin structure. They can be recruited by PtrMYB161 to its targets, *PtrWND1A/B* and *PtrMYB21*, to induce a more compact chromatin structure, which leads to the repression of *PtrWND1A/B* and *PtrMYB21* [59]. Moreover, it has been reported that *CESA4*, *IRX7/9-L/10/10-L/14*, *C4H*, *4CL1*, *HCT*, *CCOAOMT1*, *CCR1*, and *CAD1*, *SND1/3*, and *KNAT7* have a much higher overall enrichment for H3K4me3 than H3K27me3, a repression mark. Meanwhile, the orthologs of *VND1/4/5/6/7* show higher H3K27me3 signals, possibly indicating repression in fiber cells of *Eucalyptus grandis* developing xylem [33, 228].

Despite the crucial role of epigenetic modifications in regulating gene expression and responding to environmental factors, we still have limited knowledge about the epigenetic modifications of genes in the HGRNs for SCW formation. Investigation of these modifications will enhance our understanding of how DNA and histone methylation modulate the expression of genes involved in wood formation. This knowledge can shed light on how specific plant responses are induced or attenuated specific plant responses via modifying the genes in the HGRNs, ultimately leading to alternations in SCW formation to adapt to environmental changes.

### Post-transcriptional modification of the HGRNs

After genes are transcribed into pre-mRNAs, they must be processed into a mature form before translation. During this process, the production of mature mRNAs is subjected to AS, 5′ capping, and 3′polyadenalation. These modifications can increase the mRNA stability and prevent them from degeneration [229]. AS, a mechanism producing multiple transcript variants from a single gene, is a pivotal process in multicellular eukaryotes to enhance the functional diversity of the proteome [230] and play an important roles in response to environmental changes [231]. In plants, a substantial portion of mRNAs (33–60%) undergo alternative splicing [232], with over 60% manifesting as retained introns [233]. For instance, transcriptome analysis has revealed that approximately 28.3% and 20.7% of the highly expressed transcripts in developing xylem tissue undergo AS in poplar and *Eucalyptus*, respectively [234]. Taking poplar as an example, *PtrWND1B* produces two mRNA variants; one is *PtrWND1B-l* (also named *PtrSND1-A2^IR^*), which is an IR splice variant, and the other is of *PtrWND1B-s* (named *PtrSND1-A2*), which is a splice without any introns. PtrWND1B-l lacks the DNA binding and transcriptional activation domains but retains the protein dimerization domain to form heterodimers with other PtrWND members, which cannot activate their targeted genes. But, PtrWND1B-l cannot form dimers with PtrWND1B-s that has the DNA binding and transcriptional activation domains [152]. Corresponding to its functional domains, overexpression of *PtrWND1B-s* enhances fiber SCW thickening, whereas overexpression of *PtrWND1B-l* inhibits this process [67]. Similarly, *PtrWND3A/B,* and *PtrWND5A/B* (also named *PtrVND6A1/2* and *PtrVND6C1/2*) had three IR splice variants, *PtrWND3A^IR^, PtrWND3B^IR^,* and *PtrWND5A^IR^* (namely, *PtrVND6-A1^IR^*, *-A2^IR^*, and *-C1^IR^*)*,* among which PtrWND5A ^IR^ abolishes the activation function of all *PtrWNDs* except for *PtrWND5A* on their targeted genes, such as *PtrMYB21*, through forming the heterodimers [67]. These findings indicates that the AS from *PtrWND1B* and *PtrWND5A* may exert reciprocal negative cross-regulation for PtrWNDs in the HGRN for poplar SCW formation [235]. AS also occurs within other genes involved in wood formation, such as *CESA8*, *IRX6*, *LAC4/12*, *CCoMT*, *XSP1*, *XCP2*, *KNAT3*, *IAA9/11/13*, *MYB4/48/52*, *CAD4/9*, *VNI2*, *NAC061*, *ARF4/8*, and *WRKY7/33/40/44* [234], most of which belong to the HGRN for SCW formation (Figure 7). However, the biological functions of AS variants of these genes are still unknown and need further investigation.

### Post-transcriptional regulation of the HGRNs

Although up to 90% of an eukaryotic genome is transcribed into RNAs, only about 2% of the transcribed RNAs are translated into proteins [236], and the majority of remaining transcripts are non-coding RNAs (ncRNAs) [237]. Present knowledge has shown that three types of ncRNAs, microRNAs (miRNAs), long non-coding RNAs (lncRNAs), and closed circular RNAs (circRNAs), play important roles in modulating mRNA abundance and stability as well as translational efficiency [238, 239]. These mechanisms influence the functional outputs of genes, specifically the proteome contents, and functions at the nexus of plant development and environmental responses [240].

#### MiRNAs that modulate the genes in the HGRNs

MiRNAs, approximately 18–30 nt in length, are important modulators of the expression levels of a large number genes involved in nearly every aspect of plant development [241, 242]. An increasing number of miRNAs that target transcripts of genes involved in SCW formation and other processes of wood formation have been identified (Table 1). However, the present evidence appears to support that these miRNAs primarily target TFs in the HGRNs for modulation (Table 1). No miRNA has been identified to target cellulose/xylan biosynthetic genes, and only a few miRNAs that target monolignol biosynthetic genes, such as *F5H2* [243] and *C4H* [65], have been identified, indicating that miRNAs modulate SCW biosynthesis primarily through targeting TFs in the HGRNs (Figure 7). The fact that miRNAs generally do not target the SCW biosynthetic genes for modulation suggests that SCW biosynthetic genes are mainly regulated at transcriptional levels rather than by miRNAs for fast switching and there is less rate-limiting regulation in the SCW biosynthetic pathways posttranscriptionally, which may render some basal or constitutive biosynthesis of SCW components. In addition, the modulation of some upper-level TFs of the HGRNs by miRNAs can alter multiple SCW biosynthetic pathways or facilitate a switch among different SCW biosynthetic pathways to generate variable SCW components for adaptation.

**Table 1:** miRNAs that modulate the genes in the HGRNs of SCW formation

miRNA	Target gene	Function	Reference
MiR319a	*TCP4* in Arabidopsis and *PtoTCP20* in poplar	Repressing *VND7* and *PtoWND6A/B*	[34]
MiR165/166	*HB15*	Repressing *SND1* and *NST2*	[194, 244]
miR858	*MYB11/12/111*	Regulating monolignol biosynthesis	[245]
miR828	*MYB11/171*	Activating *PAL1* and *CCR2*	[35]
miR858/828	*MYB11*	Regulating monolignol biosynthesis	[35]
Novel-m0998-5p	*MYB5*	Regulating monolignol biosynthesis	[35]
miR395c	*PtrMYB2/20*	Repressing entire SCW biosynthesis-related genes	[155, 246]
miR858-x/y	*MYB83*	Repressing entire SCW formation	[49, 65]
miR858-y	*MYB35/52/63*	Repressing monolignol biosynthesis	[65, 84]
miR384	*SHN2*	Coordinately regulating SCW formation	[65, 247]
miR165-y/5168-y and miR166 family,	*HB15*	Negative regulators of monolignol biosynthesis	[65, 244]
miR165-y and miR166 families	*HB8*	Regulating monolignol biosynthesis through repressing *CCR*, *C4H*, *C3H*, and *CAD*	[65, 248]
novel-m1395-5p and novel-m0738-5p	*BEN1*	Control transcription of *CESAs* through regulation brassinosteroid levels	[65, 119]
cca-miR4391	*NAC38*	SCW formation	[249]
cca-miR11300,	*NAC103*	SCW formation	[249]
cca-miR9567-3p	*VND4*	Activating SCW formation	[249]
cca-miR9567/8689	*WRKY4*	Regulating phenylpropanoid pathway	[249]
PedmiR528-3p	*PeNST/SND1.2*	Activating entire SCW biosynthesis	[190, 250]
Ped-miR399j-5p	*PeMYB20/85.2*	Directly activating monolignol biosynthetic genes	[122, 250]
unkown_Ped-miR_44	*PeSND2/3.1* and *PeSND2/3.4*	Activating entire SCW biosynthesis	[80, 250]
*PhemiRNA159*	Overexpression inhibition the expression of *MYB33*, *NST2*, and *FRA8*	Unknow	[251]
miR6443	*F5H2*	Repressing S unit monolignol biosynthesis	[243]
novel-m0260-5p	*C4H*	Repressing monolignol biosynthesis	[65]
miR395c	*ATPS*	Indirectly down-regulating *MYB46* through reduction of Abscisic acid synthesis	[246]
69 miRNAs	unknown	Significantly different expression in the wood of low N-treated *Populus * × *canescens*	[252]*.*
198 miRNAs	Unknown	Identified in developing xylem of *Pinus massoniana*	[66]
miR156/159/166/319/396/398/408 families	unknown	Identified in xylem of rubber tree	[253]
miR393	unknown	Suppression of miR393 increases a higher expression of monolignol biosynthetic genes and a higher stem monolignol content in *Populus alba × Populus tremula var. glandulosa* unknown	[254]
miR397	17 *LACs*	Repressing monolignol polymerization of poplar xylem	[255]
miR857	*LAC7*	Regulating lignin content and consequently morphogenesis of the secondary xylem	[256]

#### LncRNAs that modulate the genes in the HGRNs

LncRNAs are noncoding transcripts longer than 200 nt and often display tissue-specific expression [257]. These molecules not only contribute to destabilization of mRNAs and repression of their translation into proteins, but also serve as targets or endogenous target mimics (eTMs) of miRNAs to reduce miRNA activity [258]. Although mounting evidence shows that lncRNAs are involved in numerous biological processes of plants [259], the study of how lncRNAs modulate genes in the HGRNs for SCW formation is still in its early stage. Up to now, there is only few lncRNAs that have been identified to regulate SCW formation. In *Populus tomentosa*, two trans-acting lncRNAs, TCONS_00060049 and TCONS_00053930, are identified to modulate *CCoAOMTs* and *4CL* in the context of tensive wood formation [260]. Another lncRNA, TCONS_00078539, is a potential target of miR168 that is implicated in participating in wood formation [261], SCW biosynthesis and auxin signaling in poplar [262]. In secondary growth of *P. tomentosa*, eight lncRNAs exhibit epistatic effects on 15 phenylpropanoid biosynthetic genes, and 28 lncRNAs are predicted to be eTMs for miRNA decoys or sponges to sequester 14 miRNAs, thereby increasing the expression of repressed target mRNAs during wood formation [262]. Notably, three lncRNAs, TCONS_00013182, TCONS00015036, and TCONS00028534, indirectly activate monolignol polymerization through interacting with ptr-miR397, which is a negative regulator of *PtrLACs* [255]. lncRNA NERDL exhibits a significant correlation with *PtoNERD* in the developmental stems of *P. tomentosa*, suggesting a common pathway involved in wood formation [263]. It is worth noting that, as of now, there is no TF in the HGRNs that has been identified to be modulated by lncRNAs to date (Figure 7).

#### CircRNAs that modulate the genes in the HGRNs

CircRNAs, a type of endogenous ncRNAs ranging from 10 to 1000 nt in length [264], exert their influence on parental gene transcription by interacting with RNA polymerase II (Pol II) [265], and they impact the translation of their target genes by acting as sponges to sequester microRNAs (miRNAs) [266], and by competing for special RNA-binding proteins [267]. Although circRNAs are widespread in plants and participate in various biological processes [264, 268], the available information on circRNAs clearly modulating genes and proteins within hierarchical HGRNs is limited (Figure 7).

It has been reported that several circRNAs influence wood properties of poplar through circRNAs–miRNAs–mRNAs regulatory chain [269]. For instance, the upregulation of circRNA1006/1344/1941/901/146 can activate *MYB61* by sponging miR5021, resulting in the higher lignin concentration in the wood. Conversely, the downregulation of circRNA1002 reduces cellulose concentrations via a circRNA1002–ptcmiR1511–CSLG3 regulatory chain in * Populus x canescens*. Furthermore, downregulation of circRNA1511/437 is implied to enhance hemicellulose biosynthesis via circRNA1511/437–ptc-miR169z–α-mannosidase regulatory chain. Finally, circRNA1226/1732/392 upregulate the expression of nuclear factor Y subunit A1-A (*NFYA1-A*), *NFYA1-B*, and *NFYA10* via modulating miR169b, which was linked to the reduced xylem width and cell layers of the xylem in poplar [269]. However, due to the challenges in characterizing the interactions of genes in these regulatory chains in a cell/tissue-specific context, there are no circRNAs that have been clearly manifested to directly regulate genes of the HGRNs for SCW formation.

### Post-translational modification (PTM) of proteins in the HGRNs

PTMs, ranging from small chemical modifications (e.g. phosphorylation) to the addition of complete proteins (e.g., ubiquitination) [270], are covalent processes that alter the localization, stability, structure, activity, and molecular interactions of the modified proteins, which is essential for growth and development [271]. In recent years, PTMs, such as phosphorylation, ubiquitination, glycosylation, and S-nitrosylation, are shown to have primordial roles in regulating the expression and function of genes in the HGRNs for SCW formation [68, 272] (Figure 7).

#### Phosphorylation modification of the proteins in the HGRNs

Protein phosphorylation, the most widespread PTM in eukaryotes [273], is critical for plants to modify multiple biological processes [274]. It has been demonstrated that phosphorylation modifies the TFs and structural genes of the HGRNs for SCW formation [68, 272, 275].

##### Phosphorylation of the TFs in the HGRNs

In Arabidopsis, Ser316Ala of NST1 can be phosphorylated in the nuclei by sucrose nonfermenting 1-related kinase 2.2/3/6 that are involved in the osmotic stress responses, resulting in changed monolignol biosynthesis in fiber cells [27]. The phosphorylation of MYB46 by mitogen-activated protein kinase 6 (MPK6), which can be activated by abiotic stresses, such as salt, cold, wounding and hyper-osmotic stresses [276], decreases its activity. In addition, the phosphorylation of MYB46 also triggers a significant degradation of MYB46 through ubiquitin-mediated proteasome pathway, leading to a substantial reduction of its transcriptional activity, and a repression of SCW formation [277]. The rapid phosphorylation of MYB46 by MPK6 followed by an extensive degradation is an efficient mechanism to regulate acute SCW formation in responses to salt stress [110]. It is notable that MYB83, a paralog of MYB46, is not phosphorylated by MPK6 [277]. BES1, a third-level TF specifically activating cellulose biosynthesis, is phosphorylated by BIN2 when BRs are at low levels, therefore promoting its degradation and inhibiting SCW formation [278]. MYB75, a negative regulator of the entire SCW formation that interacts with KNAT7 [83], can be phosphorylated by MPK4, causing an increase of its stability and a decrease of SCW formation [82]. PdLTF1, the ortholog of *MYB4* of Arabidopsis in * Populus deltoides* × *Populus euramericana*, functions as a repressor to down-regulates monolignol biosynthesis through binding the promoter of *4CL* in unphosphorylated state. After being phosphorylated by PdMPK6 in response to external stimuli such as wounding, LTF1 acts as a sensory switch to activate *4C*L, which up-regulates monolignol biosynthesis [180]. Although the phosphoproteomic analysis of stem-differentiating xylem (SDX) shows that PtrSND2/3-B1 and PtrSND2/3-B2 are also phosphorylated, the effects of these modifications on their transcriptional regulation strengths remain unclear [272].

##### Phosphorylation of the structural proteins in the HGRNs

Large-scale global phosphoproteomic analysis reveals that phosphopeptides can be mapped to 4 of 10 monolignol biosynthetic enzyme families, such as PAL, CAD, CCR, and F5H, in diverse plants [279]. In Arabidopsis, several monolignol biosynthetic enzymes, including CCR, COMT, PAL, and C4H, have potential phosphorylation sites, and phosphorylation modification is suggested to regulate their turnover or activities [280]. For example, calcium-dependent protein kinases or calmodulin-like domain protein kinase-mediated phosphorylation of PAL may be a common phosphoregulatory mechanism for its functioning in phenylpropanoid biosynthesis [281]. Moreover, protein phosphorylation can occur to PtrCesA4/7/8/17/18 and PtrIRX9 in SDX of poplar [272]. In Arabidopsis, CESA4/7/8 form a Cellulose Synthase Complex (CSC) that is essential for SCW synthesis [282]. CESA7 is phosphorylated at two serine residues in the hypervariable region between the CSC catalytic subunits, and this phosphorylation targets it for degradation [283]. Besides regulating protein stability, phosphorylation is important to regulate CESA levels via changes in CSC motility and catalytic activity [284]. In poplar, phosphorylation can mediate on/off regulation of enzyme activity for PtrF5H2, which is an important enzyme in the SDX lignification of *P. trichocarpa* [272].

These findings suggest that phosphorylation is involved in modification of many proteins within the HGRNs, however, the roles and consequences of phosphorylation of proteins in the HGRNs, particularly regarding their impact on SCW formation, need further characterization to gain a better understanding.

#### S-Nitrosylation of the proteins in the HGRNs

*S*-nitrosylation, a reversible covalent modification involving nitric oxide (NO)-related species and a cysteine residue, serves as a crucial mechanism for directly regulating cellular redox state and protein activity [285]. For example, the knockout of denitrosylase *S-NITROSOGLUTATHIONE REDUCTASE1 (GSNOR)*, which regulates protein *S*-nitrosylation by addition of a NO moiety to a cysteine thiol [286] and modulates abiotic and biotic stress responses [287], suppresses the expression of *VND7*-downstream genes and then results in lacking xylem vessel differentiation in Arabidopsis mutant seedlings, demonstrating that the knockout of *GSNOR1* disrupts VND7-mediated regulation, and GNSOR1 is a prerequisite for activating downstream genes involved in SCW [288]. However, as of now, only VND7 from the HGRN proteins is modulated by *S*-nitrosylation at present [288].

#### Ubiquitination of the proteins in the HGRNs

Ubiquitination, a common regulatory mechanism in all eukaryotes that targets proteins for degradation via the 26S proteasome, is orchestrated by a set of enzymes: ubiquitin activation enzyme (E1), ubiquitin-conjugating enzyme (E2), and ubiquitin ligase (E3) [289], This process plays crucial roles in plant growth and development as well as stress responses [290, 291]. Evidence also indicates that ubiquitination is involved in modifying the proteins in the HGRNs of SCW formation. For instance, E2 ubiquitin-conjugating enzyme 34 (PtoUBC34), induced by treatment with sodium chloride and heat shock [292], interacts with transcription repressors, such as PtoMYB221 and PtoMYB156, and translocate them to the ER, reducing their repression activity on phenylpropanoid and monolignol biosynthetic genes in a PtoUBC34 abundance-dependent manner in *P. tomentosa* [293]. The stability of VND7 is also regulated by proteasome-mediated degradation likely through interaction with RING domain protein SINA of *A. thaliana 5* [78], contributing to transcriptional homeostasis to avoid deleterious effects on xylogenesis and plant growth. AtSIZ1, a small ubiquitin-related modifier (SUMO) E3 ligase that is involved in plant growth and development [294] as well as response to various stresses by mediating sumoylation [295], mediates the sumoylation of LBD30. LBD30 positively regulates *SND1/NST1* in fiber cells [296] and *VND7* in the vessel cell [101]. The Arabidopsis Kelch domain-containing F-box (KFB) proteins (KFB01/20/39/50) that are components the E3 complex and respond differentially to environmental stimuli [297], can interact with PALs (PAL1–4), reducing PAL protein abundance by decreasing it stability [298, 299]. Double and triple mutants of *KFB01*, *KFB20*, and *KFB50* in Arabidopsis increase PAL protein abundance, resulting in more acetyl-bromide lignin in the plant cell walls. Conversely, overexpression of *KFBs* genes cause a 2 to 70% lignin reduction in the transgenic lines [299]. Small and Glossy Leaves 1, closely related to KFBs [300], can interact and reduce the stability of PAL1, leading to reduce PAL activity for monolignol biosynthesis.

### Protein–protein interactions (PPIs) of the HGRNs

Protein function can be modulated by non-covalent PPIs [301], which are frequently functionally connected with PTMs because PTMs can modulate the binding affinities between proteins [302]. PPIs can act as regulatory nodes in many cell-signaling networks and are the basis of the cellular structure and function in most biological processes [303]. It has been estimated that more than 80% of proteins do not function alone but in complexes [304]. It has been proven that the combinations of PPIs and TF-DNA interactions mainly determine the regulatory homeostasis of the HGRNs for SCW formati0n [55, 60, 136, 305] (Figure 7). Although TF–DNA interactions in the HGRNs have been extensively studied [60, 110, 305], the knowledge about PPIs is very limited. Until recently, only 165 PPIs involved in 162 different open reading frames have been identified from secondary xylem cDNA library of *P. trichocarpa* [60].

#### Interactions among the TFs in the HGRNs

Among the TFs within the HGRNs, some SWNS can bind each other to form homo-and/or hetero-dimers with different transactivation activities to regulate their downstream genes [151, 152]. For example, VND7 regulates the differentiation of all types of vessels in roots and shoots possibly through forming homodimers and heterodimers with other VND proteins (VND2 to VND5) via their N-termini, including the NAC domains [78]. Additionally, VND-INTERACTING2 (VNI2) effectively interacts with VND7 and VND1–5 at higher affinity, and other NAC domain proteins at lower affinity. Among these interactions, the VNI2 and VND7 hetero-dimer functions as a repressor of vessel-specific genes induced by VND7 [306]. During the monolignol biosynthesis in Arabidopsis, MYB4/7/32 can interact with Sensitive to ABA and Drought 2 through their GY/FDFLGL motifs, which mediates the transport of MYB4/7/32 into the nuclei and then increases the repression activity on their target genes (e.g., *4CL1/3* and *C4H*) expression [128]. The inhibition of *C4H* expression by MYB3 is also regulated by the core inhibitors, NIGHT LIGHT-INDUCIBLE AND CLOCK-REGULATED1 (LNK1) and LNK2, which act as transcriptional corepressors to facilitate binding of MYB3 to the *C4H* promoter [127]. It is interesting that KNAT7 displays complicated and spatiotemporally differentiated functions in SCW formation, depending on cell types, tissues, and its interacting partners [55]. For instance, KNAT7 can form various heterodimers with different negative regulators, such as MYB75 [83], OFP1/4 [307], and BLH6 [308], acting as a negative regulator of SCW formation in the interfascicular fiber cells [55] (Figure 3). KNAT7, OFP1/4, and BLH6 can also form a regulatory complex to repress SCW formation in Arabidopsis [136] (Figure 3). KNAT7 also interacts physically with MYB6 to repress the expression of monolignol biosynthetic genes, such as *CCoAOMT*, *CCR2*, *F5H*, *COMT2,* and *CAD1* in Arabidopsis and their homologs in poplar [164] (Figure 3 and 6). In addition, although neither KNAT3 nor KNAT7 can directly bind to the *F5H* promoter, they can form heterodimer to activate *F5H* expression in the SCW formation of xylem vessel [55] and xylary fiber cells [142]. Similarly, KNAT3 but not KNAT7 can form a heterologous complex with NST1/2 to directly activate *F5H* expression although NST1/2 cannot directly activate *F5H* expression [126]. Moreover, XND1 interacts with VND6/7 and NST1 via its C-terminal region, sequestering them in the cytoplasm, which in turn reduces their transcriptional activities in xylem differentiation [56, 57], while XND1 can interact with RBR to inhibit xylem differentiation [309]. Interaction between NST1 and XND1 likely interferes with NST1 self-interaction in formation of a homodimeric structure, which is necessary for NST1 functionality [56].

As aforementioned [153], SWNs and PtrWNDs, as the NAC TFs, can form homodimers or heterodimers with different transcriptional activities, regulating their downstream genes in the HGRNs involved in fine-tuning the regulation of SCW formation [62]. Additionally, PtrWND1B-l can form heterodimers with PtrWNDs except PtrWND1B-s and interfere with their functions in the HGRN of SCW formation [67, 152]. Similarly, PtrWND5A^IR^ interacts with PtrWNDs except PtrWND5A to act as a dominant negative regulator in the poplar HGRN [235]. It is interesting that PaC3H17 not only regulates *PaMYB199*, but also interacts with a small amount of PaMYB199 to attenuate the suppression of *PaMYB199*-regulated xylem target genes, such as *PaIRX10* and *PaIRX15L-1* involved in hemicellulose biosynthesis of *P. alba* × *P. tremula var. glandulosa* cv ‘84 K’ [58]. PaMYB4 (also named LTF1), as a key negative regulator of monolignol biosynthesis, is implied to interact with ten TFs including PaMYB21, PaDF1, PaGRAS2, and PaWRKY20 when regulating *Pa4CL3* expression [39]. PtrMYB74 can form 54 TF-TF pairs during wood formation [59, 60], among which PtrMYB74-PtrWRKY19 dimers are required to trans-activate *PtrbHLH186* and *PtrVCM2*, with *PtrbHLH186* known to regulate in monolignol biosynthesis [71]. In addition, PtrMYB74 can interact with PtrC3H18 to activate SCW biosynthesis [71], the latter positively regulates cellulose, xylan, and monolignol biosynthetic genes, and negatively regulates eight wood formation associated *MYBs* [165]. PtrMYB74 also dimerizes with PtrWOX4a/b to regulate stem cell differentiation in wood development [71, 310]. Moreover, PtrMYB74 can dimerize with three PtrWRKY family members, PtrWRKY12/13/25, as it does with PtrWRKY19 [71] (Figure 6). Furthermore, PtrMYB21-PtrMYB74, and PtrMYB90-PtrNAC123 (PtrWND1A) dimers bind to the promoters of *PtrCCoAOMT1.* PtrMYB90-PtrMYB161, PtrMYB161-PtrWBLH1, and PtrMYB90-PtrWBLH1 dimers, and PtrMYB90-PtrMYB161-PtrWBLH1 ternary complex regulate the *PtrCCoAOMT* expression level for G subunit monolignol biosynthesis [60], while PtrMYB90-PtrMYB161, PtrMYB161-PtrWBLH2, and PtrMYB90-PtrWBLH2 dimers and PtrMYB90-PtrMYB161-PtrWBLH2 ternary complex regulate *PtrF5H* abundance for S subunit monolignol biosynthesis [60].

PtrDRIF1, a MYB/SANT protein, interacts with RADIALIS (RAD) and DIVARICATA (DIV), through its N-terminal MYB/SANT domain. As a result, PtrDRIF1, can form two types of trimers, PtrDRIF1-PtrRAD1-PtrWOX13c and PtrDRIF1-PtrDIV4-PtrKNAT7, which are involved in the negative regulation of SCW formation in xylem [311]. SCL14, a key repressor encoding the DELLA protein GAI in the GA signaling pathway, interacts with NAC043 (homolog of NST1 in poplar), leading to the attenuation of the activation of NAC043 on *MYB61* in tetraploid *P. hopeiensis* stems [208]. PtoJAZ5, as an inhibitor of JA signal transduction, reduces SCW synthesis and lignin deposition through interacting with PtoWND6A and PtoMYB3 [312]. These findings suggest that interactions of TFs can increase the transcription regulation elasticity of the HGRNs for accurately regulating SCW formation.

#### Interactions among the structural proteins in the HGRNs

The interaction of one enzyme to the other can induce conformational changes that can alter enzymatic activity and substrate affinity of dimer enzyme compared to each of the two individual enzymes. It has been demonstrated that the complexes of enzymes encoded by certain structural genes in the HGRNs have been implicated to modulate SCW formation [313]. For example, CesA4, CesA7, and CesA8 interact with each other to form a CSC [112], which tracks along cortical microtubules to insert the CSC into the plasma membrane for cellulose biosynthesis [314]. During monolignol biosynthesis, membrane steroid-binding proteins serve as a scaffold to physically organize three endoplasmic reticulum (ER)-resident cytochrome monolignol P450 monooxygenases, C4H, C3H, and F5H, to establish the structural characteristics of its monomeric precursors, specifically controlling phenylpropanoid–monolignol branch biosynthesis [315]. Notably, although C4H, C3H, and F5H are in spatial proximity to each other on the ER membrane in vivo, they do not appear to directly interact with each other [315], which is not in agreement with yeast two-hybrid assay results that show the physical interactions of 4CL1 with C4H and C3H, and CCR1 with C4H [315]. However, the effects of these interactions on monolignol biosynthesis are not well understood. Additionally, C4H, C3H, and F5H also interact with two cytochrome P450 reductases (ATR1 and ATR2), where ATR2 is associated with monolignol biosynthesis and other phenylpropanoid biosynthetic enzymes, and *atr2* mutation results in a slight reduction in total lignin, potentially linked to the decreased C3H and F5H activities [316]. Moreover, C3H and C4H facilitate the association of soluble proteins PAL, HCT, and 4CL to the ER membrane, where they may form one or multiple complexes in the ER [317, 318]. Furthermore, there also are PPIs between some monolignol biosynthetic enzymes and plant defense signaling proteins. For instance, the interaction of CCR1 with Rac family small GTPase (Rac1) increases the enzymatic activation of CCR1, results in a higher accumulation of lignin in rice suspension cell cultures [319].

In poplar*,* PtrC4H1, PtrC4H2, and PtrC3H3 can form three possible heterodimers and a heterotrimer (PtrC4H1-C4H2-C3H3), which increases the reaction rates of the constituent enzyme involved in hydroxylation in monolignol biosynthesis [317]. Among these protein complexes, the PtrC4H1-C4H2 dimer facilitates cinnamic acid 4-hydroxylation, whereas PtrC4H1-C3H3 and PtrC4H2-C3H3 catalyze *p*-coumaroyl shikimic acid 3-hydroxylation, contributing to a specific 3-hydroxylation flux leading to caffeoyl shikimic acid. The trimer PtrC4H1-C4H2-C3H3 mediates both 4- and 3-hydroxylations of cinnamic acid derivatives in monolignol biosynthesis, drastically increasing enzyme metabolic efficiency [317]. In addition, two 4CL isomers, Ptr4CL3 and Ptr4CL5, form a complex that improves the homeostatic properties of CoA ligation [320], where Ptr4CL5 may play a regulatory role by affecting the kinetic behavior of Ptr4CL3 [321]. Moreover, Ptr4CL-HCT complexes modulate the metabolic flux of CoA ligation for monolignol biosynthesis during wood formation in *P. trichocarpa,* and this protein complex enhances CoA ligation activity for Ptr4CL when PtrHCT is supplemented [69]. Finally, PtrCAD1 and PtrCCR2, catalyzing the last two steps of monolignol biosynthesis, interact with each other, and their heterodimers have a higher activity than their homodimers [322].

In summary, the PPIs involving TFs and structural proteins in the HGRNs may cooperatively or combinatorically mediate the biosynthesis of specific types of SCW, which may be essential for accurate SCW formation in different developmental stages or for adaptation to environmental conditions. However, due to the lack of gene mutants in the model forest tree species, most PPIs and their specific roles in SCW formation are still unknown. However, the advent of genome editing technologies has opened up possibilities for addressing these gaps in knowledge. By employing genome editing techniques to induce mutations in individual genes or combinations of genes within the HGRNs of woody plants, such as *P. trichocarpa*, researchers can explore and uncover the intricate roles and specific regulatory mechanisms that govern SCW formation. This approach holds promise for advancing our understanding of the molecular processes underlying SCW formation and may contribute to the development of strategies for manipulating wood properties in trees for various applications.

## Conclusive marks and research focuses

Our review shed lights on many aspects of the HGRNs that govern SCW formation in the third process of wood formation, namely SCW biosynthesis and deposition, as aforementioned. The key points of our review can be recapitulated as the following:

### Conclusive marks

**4.1.1** The core HGRNs of the SCW formation in herbaceous and woody plants consist of a minimum of four hierarchical gene levels characterized by intricate regulatory relationships. Once xylem cells finish their expansion, the top-level TFs in the HGRNs detect ‘SCW formation signals’. These signals then propagate through regulatory cascades within the HGRNs, reaching the structural genes at the bottom layers. This sequence of events ultimately triggers the initiation of SCW formation.

**4.1.2** The biosynthesis of cellulose and hemicellulose is primarily regulated by the first-, first and a half-, and second-level TFs over the third-level TFs, whilst monolignol synthesis is predominantly regulated by the second-, and third-level TFs, but seldom by the first-and first and a half-level TFs.

**4.1.3** The HGRNs comprise various network motifs, such as FFLs, positive and negative FBLs spanning different levels of genes, and some positive and negative autoregulation of TFs. The most intricate and interwoven transcriptional regulatory relationships occur within the third-level TFs and their downstream target genes. The selection and combination of various regulatory cascades, chains, and network motifs of the HGRNs is the key for synthesizing heterogeneous SCW in various cells and secondary growth tissues in different developmental processes and/or various environmental conditions.

**4.1.4** Although the two HGRNs in two species have at least four layers, there are some differences in both the numbers of genes and the functions of homologous genes. It is worth noting that the SWNs and PtrWNDs have significant differences in the tissue-specific expression patterns and the regulation of PCD process.

**4.1.5** Mounting evidence in recent years indicates that the first-level TFs in the HGRNs are regulated by some TFs that are not currently integrated in the HGRNs. It is possible that these TFs are located in the ever higher hierarchical levels. Some of these TFs respond to environmental cues, suggesting that they may function as ‘signal receptors’ that connect environment cues to the HGRNs, enabling the HGRNs to be environment responsive and interactive.

**4.1.6** Among the post-translational modifications, PPIs and protein phosphorylation are currently the most extensively reported modifications within the HGRNs. These modifications have been reported across all levels of TFs, cellulose synthases, and monolignol synthases in the HGRNs, but, as of now, there is no report on the post-translational modifications within hemicellulose synthases. In contrast, ncRNAs tend to modulate TFs rather than SCW biosynthetic genes in the HGRNs.

**4.1.7** Epigenetic regulation and post-translational modifications have the capacity to incorporate the environmental signals, including various stress-responses, into SCW formation via the modifications of many genes in the HGRNs. Prioritizing research efforts to investigate these specific modifications should be a key focus for future studies.

### Future research focuses

**4.2.1** Identification of all TFs and novel regulatory relationships, especially the network motifs functioning in the HGRNs is crucial for gaining a panoramic view and a deeper understanding of the SCW formation and its regulation.

**4.2.2** Conduction of PPI analysis within the same layer of regulatory layer or across different levels of regulatory layer can lead to the identification of TFs involved in combinatorial regulation.

**4.2.3** Exploration of the spatiotemporal variation, and heterogeneous SCW formation using recently emerged technologies such as spatiotemporal single-cell RNA-seq, coupled with bioinformatics analysis, has become imperative. This approach may lead to identification of key TFs responsible for regulating tissue/cell-specific SCW formation, and these TFs could be targeted for enhancing horticultural traits and wood properties.

**4.2.4** Identification of the regulatory cascades, chains and motifs that specifically respond to ever-changing environmental stresses or factors is essential. Genetic engineering and gene editing of TFs in the same regulatory cascade, chain as well as motif simultaneously have the potential to greatly enhance a specific SCW biosynthetic pathway, and specific SCW components.

**4.2.5** The structures of the HGRNs enlighten us about how to conduct the genetic modification using CRISPR-Cas9 technology. For example, modification of a high hierarchical TF may have a broad influence on multiple SCW biosynthetic pathways, while modification of a low-level TF is likely to exclusively impact one specific pathway. Moreover, combined modifications of a high hierarchical TF, a second-level hub switch and a low hierarchical TF that are in one ‘chain-of-command’ and two combinatorial regulators (e.g., two interacting proteins) can yield diverse SCW components tailored to specific requirements.

**4.2.6.** It is imperative to identify tissue/cell-specific promoters, develop inducible promoters and ‘synthetic promoters’ that can effectively drive the genes within the HGRNs. This is particularly crucial when aiming to improve most horticultural traits that requires localized genetic modifications through genetic engineering.

## Data Availability

There is not data available.
